# A Non-Canonical Initiation Site Is Required for Efficient Translation of the Dendritically Localized Shank1 mRNA

**DOI:** 10.1371/journal.pone.0088518

**Published:** 2014-02-12

**Authors:** Katrin Studtmann, Janin Ölschläger-Schütt, Friedrich Buck, Dietmar Richter, Carlo Sala, Jürgen Bockmann, Stefan Kindler, Hans-Jürgen Kreienkamp

**Affiliations:** 1 Institut für Humangenetik, Universitätsklinikum Hamburg-Eppendorf, Hamburg, Germany; 2 Institut für klinische Chemie; Universitätsklinikum Hamburg-Eppendorf, Hamburg, Germany; 3 Zentrum für Molekulare Neurobiologie Hamburg, Universitätsklinikum Hamburg-Eppendorf, Hamburg, Germany; 4 Consiglio Nazionale delle Ricerche, Institute of Neuroscience, Milano, Italy; 5 Institut für Anatomie; Universität Ulm, Ulm, Germany; CNRS UMR7275, France

## Abstract

Local protein synthesis in dendrites enables neurons to selectively change the protein complement of individual postsynaptic sites. Though it is generally assumed that this mechanism requires tight translational control of dendritically transported mRNAs, it is unclear how translation of dendritic mRNAs is regulated. We have analyzed here translational control elements of the dendritically localized mRNA coding for the postsynaptic scaffold protein Shank1. In its 5′ region, the human Shank1 mRNA exhibits two alternative translation initiation sites (AUG^+1^ and AUG^+214^), three canonical upstream open reading frames (uORFs1-3) and a high GC content. In reporter assays, fragments of the 5′UTR with high GC content inhibit translation, suggesting a contribution of secondary structures. uORF3 is most relevant to translation control as it overlaps with the first in frame start codon (AUG^+1^), directing translation initiation to the second in frame start codon (AUG^+214^). Surprisingly, our analysis points to an additional uORF initiated at a non-canonical ACG start codon. Mutation of this start site leads to an almost complete loss of translation initiation at AUG^+1^, demonstrating that this unconventional uORF is required for Shank1 synthesis. Our data identify a novel mechanism whereby initiation at a non-canonical site allows for translation of the main Shank1 ORF despite a highly structured 5′UTR.

## Introduction

Local protein synthesis is a cellular mechanism to generate an asymmetrical protein distribution in polarized cells. Neurons employ this mechanism to provide specific newly synthesized proteins to dendritic segments, spines or postsynaptic sites [1]. Local changes in protein composition can occur e.g. at recently activated synapses as a prerequisite to the so-called input specificity of synaptic plasticity [2]. This model requires that certain mRNAs are indeed present in dendrites in significant amounts, and that these messages will only be translated upon the appropriate stimulus, while otherwise being translationally silent.

So far it is mostly unclear how translation of dendritic mRNAs is regulated. A number of studies have analyzed signalling pathways which may stimulate translation in dendrites (e.g. [3]). In particular, it has been suggested that activation of metabotropic glutamate receptors (mGluRs) activates synaptic protein synthesis, and that this process is inhibited by the RNA binding fragile X mental retardation protein (FMRP; [4], [5], [6]). Also, a role for the mTOR/PKB pathway has been suggested [7]. However, it is clear that besides a number of *trans*-acting factors, *cis*-acting sequence elements must exist on dendritic mRNAs, which enable control by these cellular pathways.

For most mRNAs, initiation of translation involves the 5′end recruitment of the 43S initiation complex containing of the small ribosomal subunit and different eukaryotic initiation factors [8]. This complex then scans the 5′-untranslated region (UTR). When it recognizes an AUG initiation codon in a favorable context (so-called Kozak sequence), the 60S ribosomal subunit joins the complex to form an elongation-competent 80S ribosome. This will enable most scanning 43S complexes to initiate translation at this position [9], [10]. Stable secondary structures may stop the scanning of the 43S complex and thereby restrain translation initiation [11]. Similarly short upstream open reading frames (uORFs) located in the 5′UTR may reduce the initiation rate at the authentic start codon (AUG^+1^) that precedes the main open reading frame (ORF; [12], [13]). Ribosomes finishing translation of a uORF may either completely detach from the mRNA or the 40S subunit may resume scanning and initiate translation at a downstream AUG, leading to ORF translation. The reinitiation rate depends on a number of parameters such as the uORF length, and the distance and sequence between the start codon of the uORF (uAUG) and the downstream initiation site. However, when uORF and ORF overlap ribosomes that translate the uORF will move past AUG^+1^ and will thus not translate the complete ORF. Therefore, only 43S complexes which skip the uAUG will be able to initiate translation at AUG^+1^. Interestingly, we have recently shown that such a mechanism contributes to formation of two protein variants translated from the dendritically localized SAPAP3 mRNA [14].

In the past we have started to analyze translational control elements within the 5′UTR of the mRNA coding for the postsynaptic scaffold protein Shank1. The Shank1 mRNA is particularly abundant in dendrites of hippocampal neurons and cerebellar Purkinje cells [15], [16]. Shank family members (Shank1-3, also termed SSTRIP/ProSAP) regulate the morphology of dendritic spines [17] and significantly contribute to the formation and stabilization of the postsynaptic density (PSD; [18]). Loss of function allels in all three *SHANK* genes have been associated with either mental retardation or autism in humans [19], [20], [21]. On the other hand, translation of the Shank1 mRNA is inhibited by the fragile X mental retardation protein (FMRP), and FMRP deficient mice exhibit elevated postsynaptic Shank1 levels [22], [23]. Taken together, these data suggest that Shank levels at postsynaptic sites need to be precisely controlled. We have previously shown that translation of the Shank1 mRNA is inhibited by its 5′UTR. In contrast to other dendritically localized transcripts, Shank1 mRNA translation is not driven by an internal ribosomal entry site (IRES; [24]). Two features of the Shank1 5′UTR might contribute to translational control: (i) the extremely high GC content which could lead to formation of stable secondary structures, interfering with ribosomal scanning, or (ii) several overlapping uORFs, which might interfere with access of scanning ribosomes to AUG^+1^ of the Shank1 ORF. By analyzing the contribution of individual uORFs to translational control, we demonstrate that an unusual (non-AUG) translation initiation site is required to maintain translation of the Shank1 mRNA at a basal level.

## Results

### Translational control elements in the human Shank1 5′UTR

**Figure 1:**
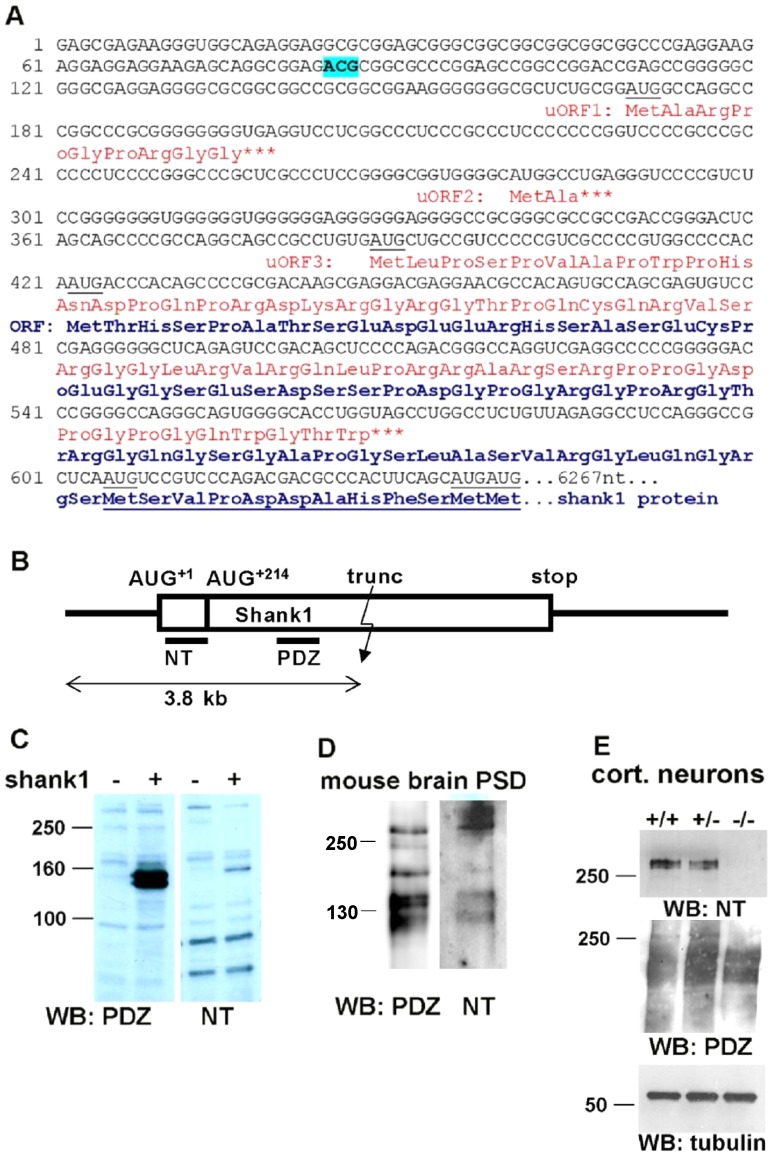
Alternative translational start sites in the Shank1 5′UTR. A. Sequence of the 5′ region of the human Shank1 mRNA; seven possible AUG start codons are underlined. Translation initiation at the first three of these leads to premature termination of translation (uORFs 1–3; translation products labeled in red). AUG^+1^, and a cluster of three closely appositioned AUGs (collectively termed AUG^+214^) may both initiate translation of the full-length Shank1 ORF. Start at AUG^+1^ leads to an N-terminal elongation of the Shank1 protein by 70 amino acids. Amino acid sequence common to both variants is underlined. An ACG codon (nt 84–86) which becomes important later in this manuscript is indicated. B. Scheme of the Shank1 mRNA; UTRs are indicate by a line, the coding region is boxed. In frame AUGs of the main ORF, the size of a truncated expression construct of 3.8 kb, and the position of different antigenic domains (NT, PDZ) used for raising antisera are indicated. C. HEK cells were transfected with no plasmid (−) or with the truncated Shank1 expression construct (+) encompassing the first 3800 bp of the Shank1 cDNA, including the complete 5′UTR. Cell lysates were analyzed by Western blotting using an antiserum directed against the PDZ domain (left), or against the alternative N-terminal region generated by initiation at AUG^+1^ (NT; right). Note that the PDZ antibody recognizes two bands at approximately 130–140 kDa (calculated: 116 and 123 kDa), whereas the NT antibody recognizes only the upper one of these two bands. D. A PSD preparation derived from mouse brain was analyzed by Western blotting with PDZ and NT directed antibodies. E. Cortical neurons were prepared from wt (+/+), heterozygous (+/−) and Shank1 deficient (−/−) mice. Cells were lyzed and analyzed by Western blotting using anti-Shank1-NT (upper), anti-PDZ (middle) and anti-tubulin (lower panel) antibodies.

The human Shank1 mRNA contains a long 5′UTR of 421 bases [24] see Fig. 1A) that harbors three uORFs (uORF1-3 in Fig. 1A). 213 nucleotides downstream of AUG^+1^ of the Shank1 ORF lies a cluster of three AUGs (collectively termed AUG^+214^ here). Both AUG^+1^ and AUG^+214^ are possible initiation sites which are conserved in rat, mouse and human Shank1 mRNA [24]. These start sites could lead to synthesis of distinct mature Shank1 isoforms. To assess whether both putative initiation sites may be used, a partial human Shank1 mRNA, encompassing only the first 3.8 kb (including all known 5′UTR sequences) was expressed in HEK 293 cells. This gave rise to two distinct protein species around 140 kDa which were both detected by an antibody recognizing the PDZ domain present in all known Shank1 variants (anti-PDZ; Fig. 1B,C). The difference in molecular weight between these species might well be accounted for by the 71 amino acid residue difference of the two predicted translation products (calculated: 116 kDa/123 kDa for the smaller and larger form, respectively). We raised an antiserum against the additional N-terminal protein sequence (termed NT domain) encoded by the mRNA between AUG^+1^ and AUG^+214^. This antiserum (anti-NT) recognized only the higher molecular weight band. In Western blots performed with mouse brain PSD preparations (Fig. 1D) and lysates of mouse cortical neurons (Fig. 1E) anti-NT specifically recognized a protein of more than 250 kDa, while anti-PDZ recognized different Shank1-3 isoforms between 180 kDa to slightly above 250 kDa in the PSD fraction (Fig. 1D). The largest band identified by anti-NT is absent in neurons derived from Shank1 deficient mice (Fig. 1E), further confirming its identity as a Shank1 protein variant. We can not exclude at this stage whether the smaller of the two bands in Figure 1C is caused by partial degradation of the larger protein. However, as the shift in molecular weight between the two forms observed here matches quite well to the calculated difference caused by the additional 71 amino acids, we favour the interpretation of two translational start sites. It should be noted that a similar situation exists in the mRNA coding for rat ProSAP2/Shank3 (GenBank: AJ133120.1), where the predicted longer translation product carries an N-terminal extension of 75 amino acids.

### Functional analysis of the Shank1 5′UTR

**Figure 2:**
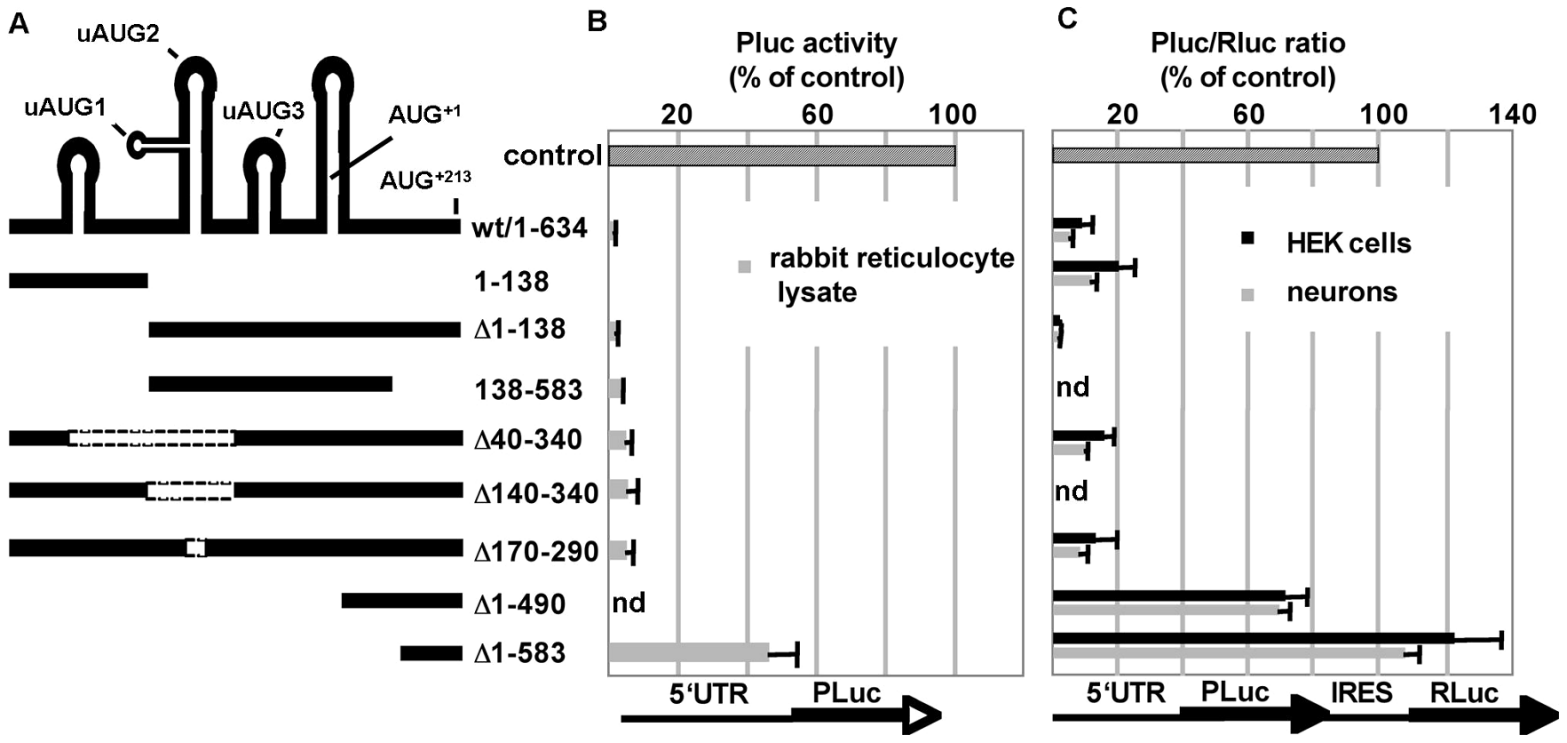
Deletion analysis of the 5′UTR of the Shank1 mRNA. A. Schematic representation of the Shank1 5′UTR; the upper panel indicates segments with extensive secondary structure, as determined using the program mfold; AUG start codons are indicated. Deletions were introduced as indicated. B. cDNA fragments were cloned upstream of the Photinus luciferase coding sequence (control: no insert). Capped transcripts were generated, and translated *in vitro* using rabbit reticulocyte lysates; these were then assayed for luciferase activity to determine translation efficiency (mean +/−SD; n = 3). C. cDNA fragments were introduced into a bicistronic vector, consisting of (5′ to 3′): a CMV promoter, the cDNA fragment of interest, Photinus luciferase coding sequence, an IRES element derived from EMCV, Renilla luciferase coding sequence. Constructs were transfected into HEK cells (black) and cortical neurons (grey). Photinus and Renilla luciferase activities (Pluc; Rluc) were recorded, and translational efficiency is depicted as the Pluc/Rluc ratio (mean +/−SD; n = 5).

We have described before that the Shank1 5′UTR reduces the translation rate to less than 10% of control values [24]. To further assess how this repression is achieved we evaluated the *in vitro* translation efficiency of recombinant mRNAs containing the first 634 nt of Shank1 mRNAs (up to, and including AUG^+214^) fused in frame to the *Photinus* luciferase coding region in rabbit reticulocyte lysates.Translation was strongly repressed by the Shank1 5′UTR. Successive deletions within the 5′UTR led to a stepwise increase in luciferase levels only when large portions of the 5′ region were deleted (Fig. 2A,B). To verify these findings in a cellular context we used bicistronic reporter mRNAs, which are essentially identical to the recombinant transcripts described above, but additionally contain the EMCV IRES and *Renilla* luciferase coding region downstream of the *Photinus* sequence. Both *Photinus* and *Renilla* luciferase activities were determined in lysates of transfected HEK cells and cortical neurons. As both luciferases are encoded by the same transcript, changes in the activity ratio are due to alterations in translation initiation efficiency controlled by the Shank1 5′UTR. In both cell types, translation rates approached control (vector encoded short 5′UTR) values only when more than 400 bases were deleted from the 5′ end of the reporter mRNA. Interestingly, as compared to the complete 5′UTR, deletion of the first 138 nucleotides led to a further reduction in translation efficiency. On the other hand, these 138 nucleotides alone inhibited translation to an extent comparable to that of the full length 5′UTR (Figure 2C). These data indicate that more than one part of the Shank1 5′ UTR may exert an inhibitory influence on translation.

### The role of uORFs in the Shank1 5′UTR

**Figure 3:**
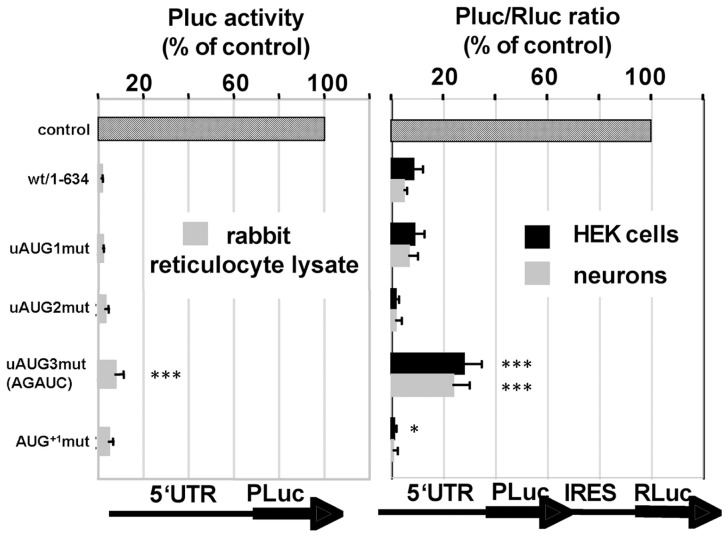
Mutational analysis of upstream open reading frames. Individual upstream start codons of the Shank1 5′UTR were mutated to AAG, and corresponding cDNA fragments were fused to the Photinus luciferase (Pluc) coding region as described in the legend to Figure 2. For mutation of uAUG3, two bases were exchanged, changing the sequence from UGAUG (wt) to AGAUC (uAUG3mut). Translational efficiency was analyzed by *in vitro* translation of capped transcripts, using rabbit reticulocyte lysates (left) or in transfected HEK cells and primary cultured cortical neurons by transfecting bicistronic vectors (right), as indicated. *(***), significantly different from wt 5′UTR, p<0.05 (p<0.001); ANOVA, followed by Dunnett's test for multiple comparisons.

To analyze the role of the uORFs for translation efficiency, uAUG1-3 were individually disabled by point mutation within the context of luciferase reporter vectors and assayed as before. Inactivation of uORF1 or uORF2 (both of which reside entirely within the 5′UTR) did not significantly alter luciferase activity, neither *in vitro* nor in a cellular context (Fig. 3A,B), indicating that both uAUG1 and uAUG2 do not contribute to translation control. For technical reasons, inactivation of uAUG3 was obtained by two point mutations, one changing AUG3 to AUC and a second leading to an U to A exchange further upstream (changing UGAUG to AGAUC; see Fig. 3; also see below). When compared to the wt 5′UTR, the AGAUC mutation caused a slight increase in translation efficiency (Fig. 3). Mutation of AUG^+1^ reduced translation efficiency in transfected cells, indicating that AUG^+1^ may be used efficiently.

**Figure 4:**
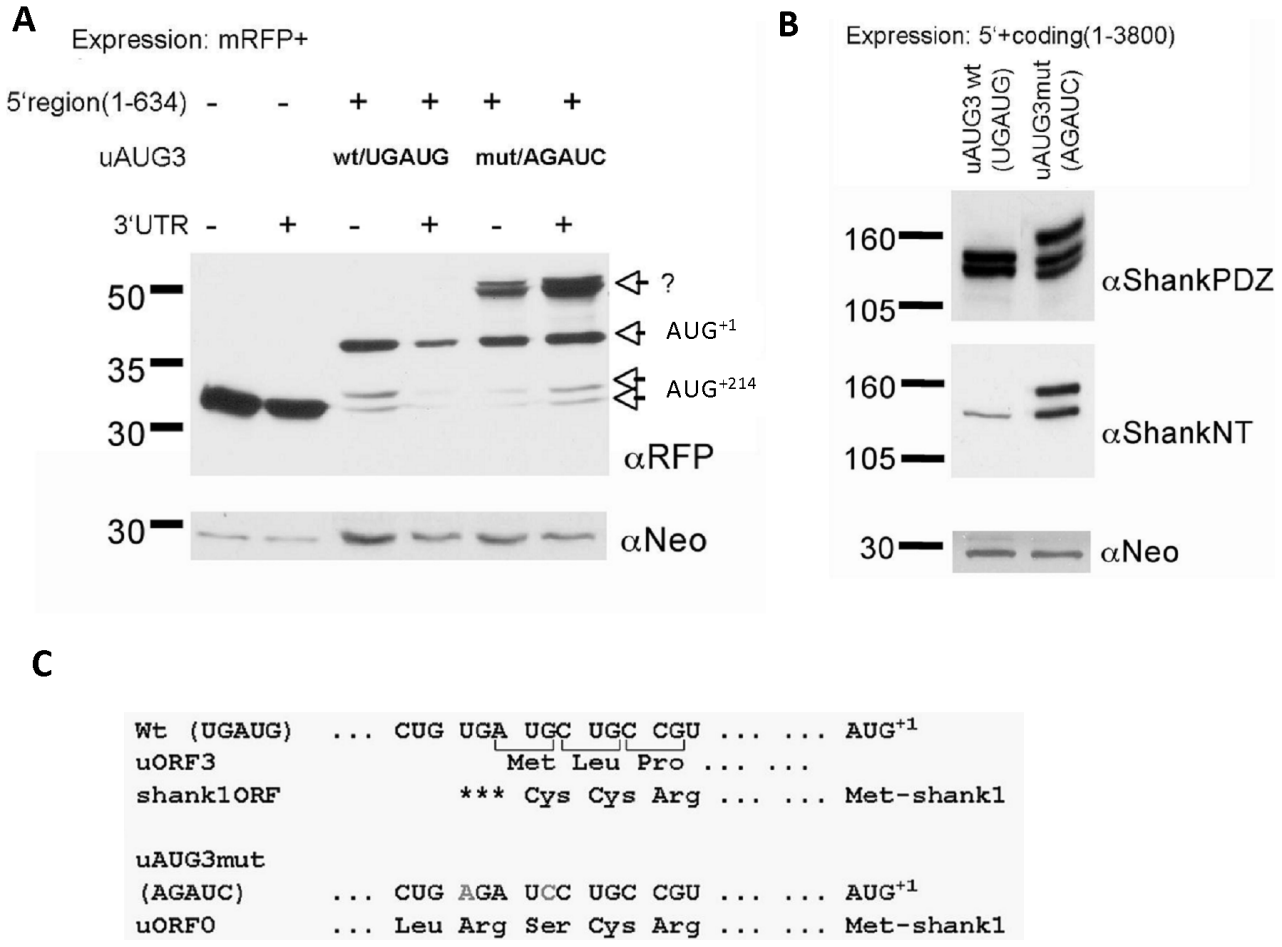
Consequences of mutation of uAUG3. A. Fusions of the mRFP coding region with wt or uAUG3 mutant Shank1 5′UTR and in some cases also a partial Shank1 3′UTR were expressed in HEK cells; cell lysates were analyzed by Western blotting using anti-mRFP. The positions of predicted translation products initiated at AUG^+1^ and AUG^+214^ are indicated; a novel, higher molecular weight protein is indicated by a question mark. All blots were reprobed with an antibody against the product of the neomycin resistance gene (neo) encoded by the expression vector, to ensure similar transfection efficiency. B. A 3.8 kb cDNA fragment containing a large portion of the Shank1 coding region and either wt or uAUG3 mutant 5′UTR was expressed in HEK cells. Cell lysates were analyzed by Western Blotting using anti-PDZ or anti-NT antibodies. Note that the additional higher molecular weight isoform observed with the uAUG3 mutant construct is recognized by both antibodies. Blots were again reprobed with anti-neo. C. Scheme of open reading frames occurring in wt and uAUG3 mutant Shank1 5′UTR. In the wt sequence two ORFs are possible; uORF3 (indicated by brackets) is not in frame with the Shank1 main ORF. For generating uAUG3mut, two bases had to be exchanged which are depicted in grey (change from UGAUG to AGAUC); whereas uAUG3 is lost (AUG to AUC), the Shank1 open reading frame is extended in the 5′ direction due to the loss of the in frame stop codon (UGA to AGA).

uORF3 terminates downstream of AUG^+1^. Thus, initiation at uAUG3, followed by translation reinitiation at AUG^+214^ will lead to the synthesis of the short form of Shank1 lacking the NT region. To analyze whether inactivation of uORF3 might affect the ratio between both Shank1 isoforms (see Fig. 1C), reporter mRNAs containing the mRFP coding sequence were expressed. In the absence of Shank1 5′UTR, mRFP was detected at approx. 32 kDa using an mRFP-specific antibody. Addition of the Shank1 5′UTR to the expression construct led to two weak bands around 32 kDa, and a stronger band at 38 kDa, consistent with translation initiation at AUG^+1^ (38 kDa) and at the cluster of three AUGs at position +214 (32 kDa). Surprisingly, the mutation of uAUG3 (in terms of the AGAUC double mutation) led to prominent synthesis of a 52 kDa recombinant mRFP protein in addition to the 32 and 38 kDa products translated from the non-mutated reporter mRNA (Fig. 4A). In contrast to the Shank1 5′ UTR, addition of the 3′UTR to expression constructs did not affect protein production.

Similar to the effect seen with mRFP reporter constructs, introduction of the uAUG3/AGAUC mutation into the 3.8 kb truncated Shank1 mRNA (which normally leads to synthesis of two 130 and 140 kDa Shank1 fragments; see above, Fig. 1) also gave rise to an additional 160 kDa translation product (Fig. 4B).

Generation of these larger protein species from uAUG3 mutant mRNAs could not be explained by any upstream AUG start codon present in the Shank1 5′ UTR. Therefore we reexamined our expression constructs; as mentioned above, in the AGAUC mutant, the additional point mutation upstream of uAUG3 had to be introduced for cloning reasons. This mutation converts the putative UGA stop codon overlapping with uAUG3 into an amino acid encoding triplet (AGA) that is in frame with AUG^+1^ (Fig. 4C). Our data suggest that this sequence alteration creates an artificial long ORF in the reporter transcripts that starts with an initiation codon located upstream of AUG^+1^(in fact upstream of uAUG3) and encodes an N-terminally extended recombinant protein. However, no potential in-frame AUG start codon was identified upstream of AUG^+1^, indicating that a non-conventional initiator site leads to synthesis of the additional long reporter protein.

**Figure 5:**
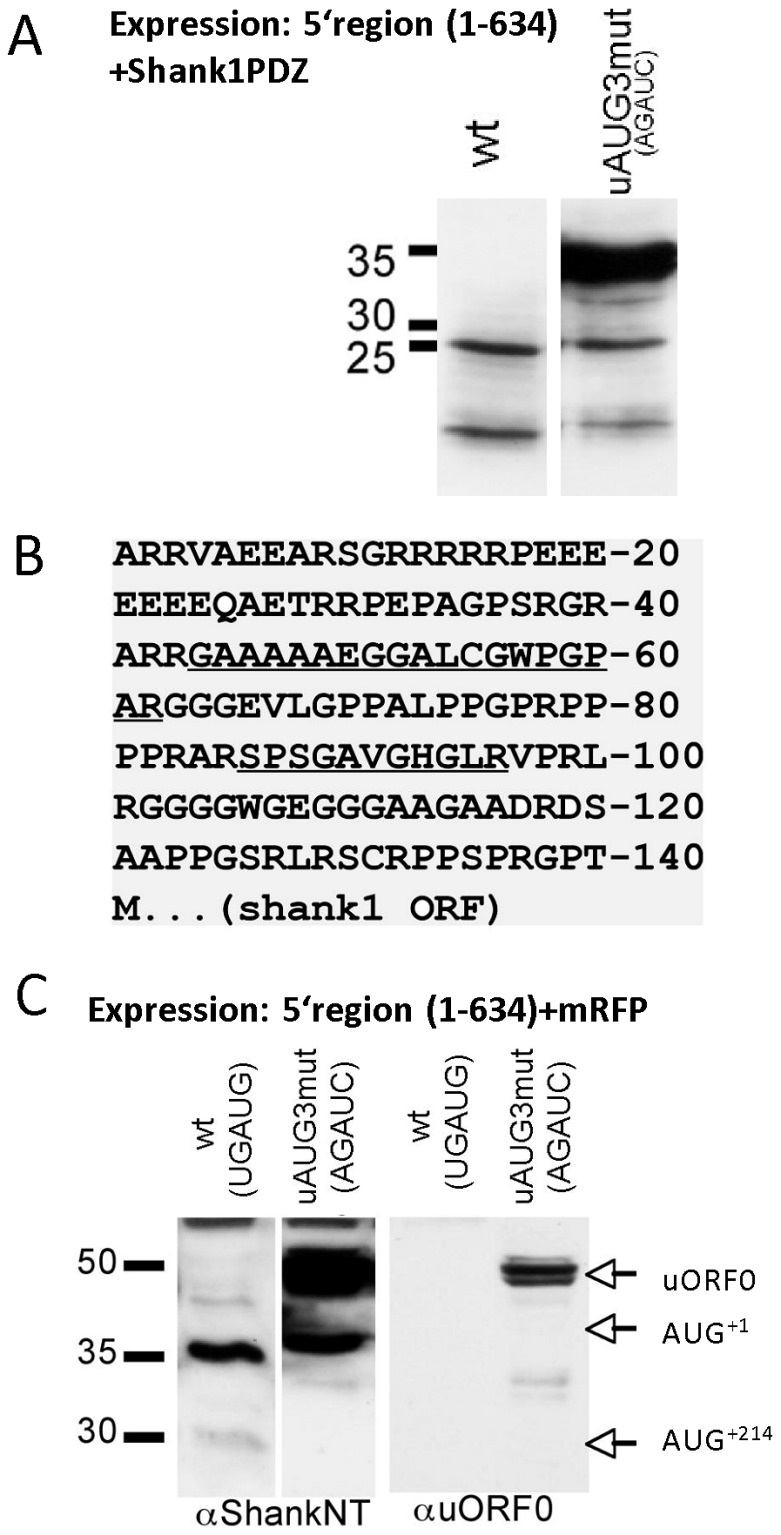
Identification of an N-terminally extended open reading frame. A. cDNAs coding for WT and uAUG3 mutant Shank1 5′UTR were fused to sequence coding for the Shank1 PDZ domain. The resulting expression constructs were transfected into HEK cells, and cell lysates were analyzed by Western blotting with anti-PDZ domain antibody. B. Predicted protein translation of the 5′UTR/uAUG3 mutant mRNA in frame with the AUG^+1^ start codon. For identification of this protein, the uAUG3 mutant/PDZ fusion construct described in A was expressed in HEK cells, and PDZ containing proteins were affinity purified by affinity chromatography using immobilized GKAP C-terminal peptide. After SDS-PAGE analysis, the prominent band at 50 kDa (see A) was cut out and analyzed by mass spectroscopy. Non-PDZ domain peptides identified in this analysis are underlined. C. Fusions of the mRFP coding region with wt or uAUG3 mutant Shank1 5′UTR were expressed in HEK cells and analyzed by Western blotting using anti-NT and an antiserum directed against the translation product of the novel open reading frame (uORF0) depicted in B.

To confirm this biochemically, we fused wt and uAUG3/AGAUC mutant 5′UTR to sequence coding for the Shank1 PDZ domain only. This short protein can be easily purified from cell extracts using the C-terminal peptide of SAPAP1/GKAP as an affinity ligand [25]. Again, an increased molecular weight of the expressed protein (35 kDa compared to bands at 22 and 15 kDa for the PDZ domain construct containing wt 5′UTR) was obtained when the mutation was introduced (Fig. 5A). The resulting 35 kDa protein was purified by GKAP chromatography, subjected to SDS-PAGE and the 35 kDa band was excised from the gel. Tryptic peptides were then analyzed by mass spectroscopy. Thereby, we unequivocally identified several peptides that are encoded by the putative ORF located upstream of AUG^+1^ (Fig. 5B).

We raised an antiserum against the peptide encoded by the putative upstream ORF; after expression of reporter mRNAs containing wt or mutant 5′UTR fused to mRFP, this antiserum detected the 50 kDa reporter protein, but not the two shorter translation products in lysates of transfected HEK cells.These data confirmed the results of the mass spectroscopic analysis (Fig. 5C). Taken together, our findings clearly indicate that in addition to uORF1-3 the Shank1 5′UTR contains a further uORF (termed uORF0 here) that starts with a non-conventional initiator site. This upstream ORF ends with an UGA stop codon overlapping with uAUG3 (in the UGAUG wt Shank1 5′UTR), but extends into the main ORF of Shank1 in AGAUC mutant constructs.

**Figure 6:**
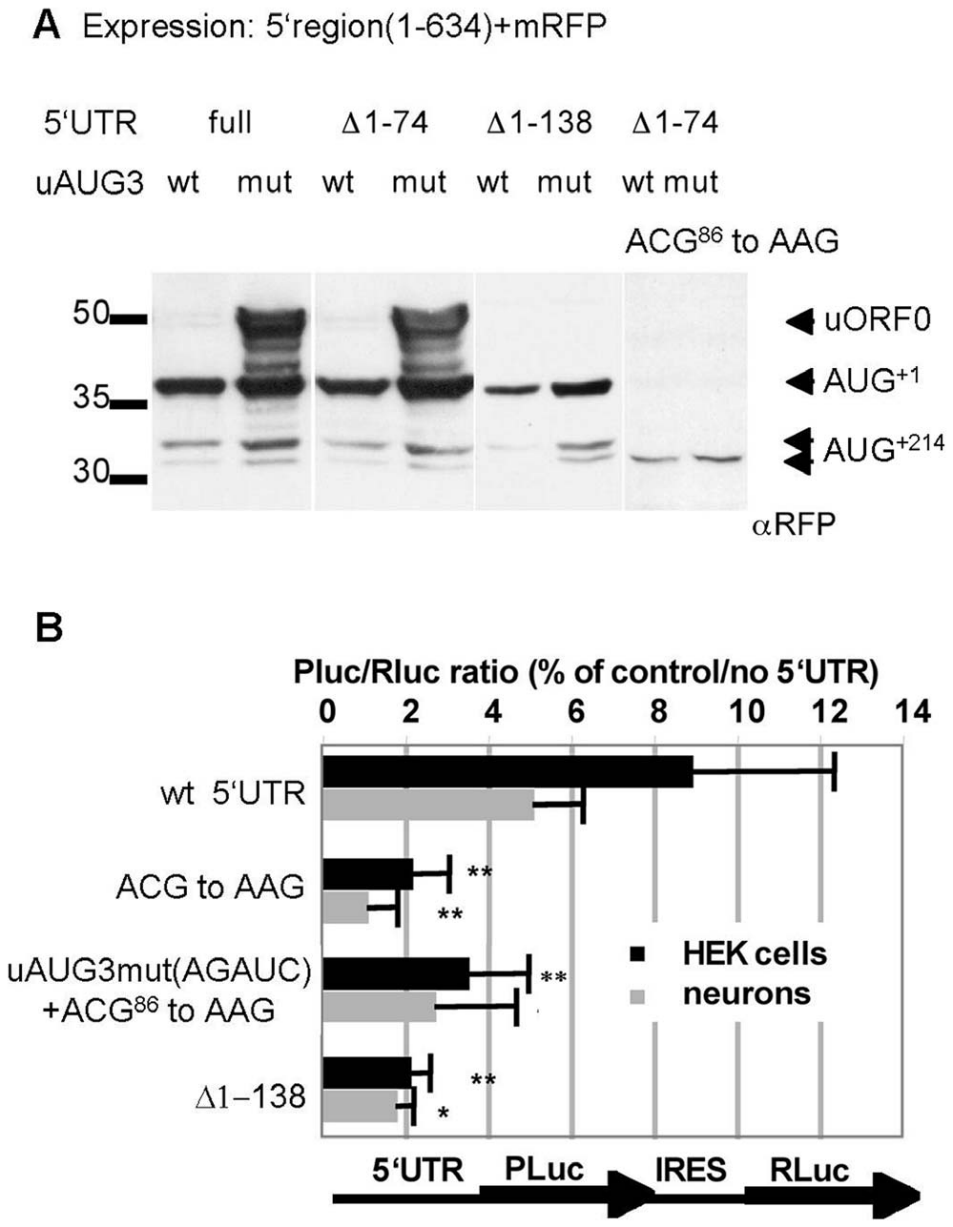
Identification of an unconventional start codon in the Shank1 5′UTR. A. The mRFP coding region was fused with wt or uAUG3 mutant (AGAUC) Shank1 5′UTR; to map the translation start site, stepwise deletions of 74 and 138 bases were introduced, as well as a mutation of nucleotides 84–86 (ACG to AAG). All constructs were expressed in HEK cells and cell lysates were analyzed by Western blotting using anti-mRFP. B. Bicistronic expression vectors containing the indicated mutant versions of the Shank1 5′UTR were expressed in HEK cells and primary cultured neurons as indicated, and analyzed for the activities of the expressed luciferases as described in Figures 2 and 3. Data are presented as the percentage of the Pluc/Rluc ratio obtained with the control plasmid lacking any Shank1 5′UTR sequence. * (**), significantly different from wt 5′UTR, p<0.05 (0.01); ANOVA, followed by Dunnett's test for multiple comparisons; n = 3.

Next, we attempted to identify the non-canonical initiation codon of uORF0. The molecular weight of the expressed reporter proteins and the position of the most N-terminally located tryptic peptides (Fig. 5B) suggested that the uORF0 initiation site lies between nt 1–138 of the Shank1 5′UTR. Deletion of the first 138 but not 74 nt from 5′UTR-mRFP reporter transcripts carrying the AGAUC mutation interfered with the synthesis of the recombinant 50 kDa mRFP variant (Fig. 6A), thus positioning the uORF0 start codon downstream of nt 74. Using start site prediction software (NetStart and ATGpr; see Methods) we identified an ACG codon (nt 84–86, coding for Thr) as the most likely translation initiation site between nt 74 and 138. In agreement with this assumption, the exchange of this ACG codon for a non-initiator site (AAG) in AGAUC mutant transcripts led to a complete loss of the 50 kDa, uORF0 derived protein. In addition, initiation at AUG^+1^ was lost in both the wt (UGAUG) and the AGAUC mutant transcripts (Fig. 6A). Only translation initiating at AUG^+214^ prevailed at a low level. These data show that the uACG at nt 84–86 indeed serves as uORF0 start site and that the use of uORF0 enhances translation initiation at AUG^+1^. In Shank1 mRNAs uORF0 overlaps with uORF3, which again overlaps with the Shank1 ORF (Fig. 1). Thus, ribosomes translating uORF0 will bypass uAUG3 and are therefore capable to reinitiate translation at AUG^+1^ to synthesize Shank1-long. However, skipping of the uACG by the 43S complex may result in the translation of uORF3. These ribosomes will bypass AUG^+1^ and may therefore reinitiate translation at AUG^+214^ leading to the synthesis of the shorter form of Shank1. In agreement with this notion, mutation of the uACG in Photinus luciferase reporter transcripts harboring the wildtype Shank1 5′UTR strongly diminished luciferase activity in HEK293 cells and in primary cultured neurons (Fig. 6B).

## Discussion

To obtain further insight into translational regulation of Shank1 mRNAs, we analyzed the contribution of the 5′UTR to translation control in both a human cell line as well as in cultured hippocampal neurons. Our data show that (1) Shank1 translation is controlled by a long 5′UTR which is GC rich and contains several uORFs; (2) overlapping uORFs enable the synthesis of two different forms of Shank1; and (3) a non-canonical initiation site is necessary to allow for low levels of Shank1 mRNA translation.

One major reason for low translation efficiency of reporter transcripts may be the high GC content of the 5′UTR. GC rich sequences can form stable secondary structures, which interfere with scanning of 43S complexes from the 5′ cap to either AUG^+1^ or AUG^+214^. It was shown recently that specific RNA helicases are used to overcome such an inhibition [26]. We did not specifically address the relevance of secondary structures here. However, we note in our deletion analysis (Fig. 2) that several smaller fragments of the 5′UTR are able to reduce translation efficiency, suggesting that each of these fragments may, possibly due to its GC content, provide a significant obstacle for the scanning 43S complexes.

The Shank1 mRNA bears similarities to the dendritically localized SAPAP3 transcript, as synthesis of the SAPAP3 protein is also affected by the high GC content of the 5′UTR. In addition, the SAPAP3 message contains an uORF which extends into the main ORF, thereby directing synthesis of two different mature forms of the SAPAP3 protein [14].

**Figure 7:**
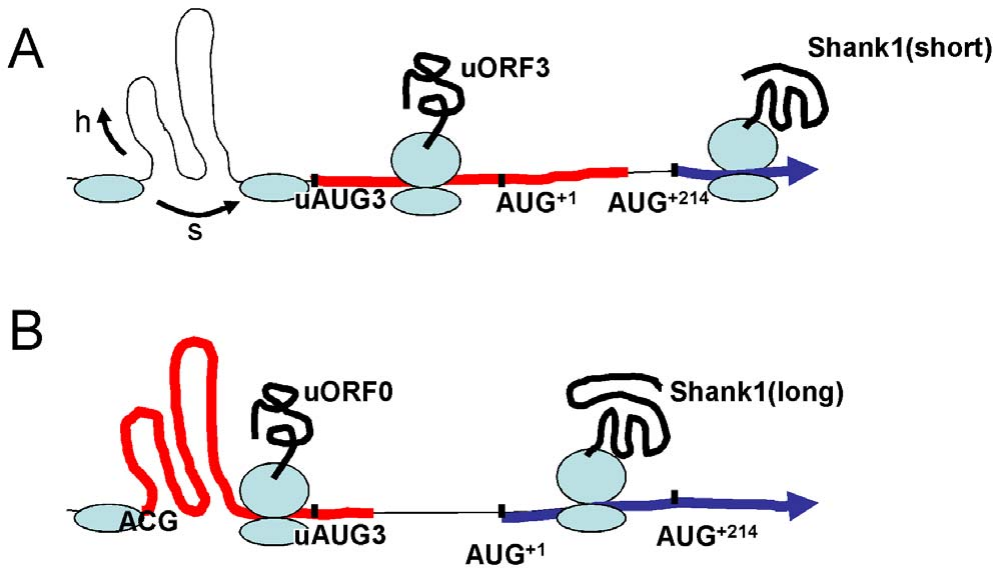
Model of the alternative use of upstream open reading frames in the Shank1 mRNA. A. In the Shank1 5′UTR, the scanning 40S subunit of the ribosome encounters strong secondary structure, blocking access to translation start sites. These difficulties may be overcome either with the help of associated RNA helicase activities (h arrow) or by a shunting mechanism (s arrow). After reaching uAUG3, uORF3 (red) is translated and reinitiation at AUG^+214^ leads to synthesis of the short form of the mature Shank1 protein (blue). B. Alternatively, the non-canonical ACG start codon is used; uORF0 (red) is translated, allowing the 80S ribosome to proceed beyond uAUG3. Reinitiation occurs at AUG^+1^, leading to synthesis of the long version of Shank1 (blue).

We observe here that in our Shank1 reporter mRNAs, uORF1 and uORF2 do not affect translation efficiency, whereas uORF3 plays a regulatory role as it allows for discrimination between synthesis of the short and the long forms of Shank1 identified here. Despite the high GC content of the upstream region of the Shank1 mRNA, uAUG3 may be reached with the help of RNA helicases or through a ribosomal shunting mechanism (see model in Fig. 7; [27]). Ribosomes translating uORF3 will bypass AUG^+1^. 40S subunits that resume scanning thereafter may reinitiate translation at AUG^+214^, leading to the synthesis of Shank1-short. A similar mechanism has recently been observed to be relevant *in vivo* in case of the C/EBPb protein in hepatocytes [13].

This leads to the question how synthesis of Shank1-long, initiated at AUG^+1^, may occur at all, given that access of 43S scanning complexes to this start site is blocked by high GC content and uAUG3. This is where our discovery of the non-canonical ACG start site becomes relevant, which may be used to initiate translation of uORF0 (see Fig. 7B). The higher processivity of translating ribosomes, compared to scanning 43S initiation complexes will enable the ribosomes reading uORF0 to proceed through the highly structured central portion of the 5′UTR and thereby move past uAUG1 and uAUG2. As the stop codon of uORF0 coincides with uAUG3, post-termination 40S subunits will only resume scanning downstream of uAUG3 and may initiate translation at the next downstream start codon, namely AUG^+1^, thereby leading to the synthesis of Shank1-long (Fig. 7B). Consistent with this model, ACG mutant or Δ138 versions of our reporter transcripts led to complete loss of translation products initiated at AUG^+1^. In this model, the non-canonical ACG start codon is necessary to maintain the low level of translation, which we see in our experimental systems, and together with uAUG3 determines start site selection.

Our discovery of a non-canonical upstream start codon raises the question whether translation initiation at non-canonical start codons may be a more widespread phenomenon. Interestingly, it was recently shown that synthesis of ornithine decarboxylase homologs in mammals is regulated by the peptide encoded by an evolutionary conserved uORF starting with a AUU codon [28]. As the translation product of Shank1 uORF0 is not highly conserved across species, we have not considered this possibility in our case. Non-canonical start sites have been demonstrated in a number of mRNAs where they lead to an N-terminal extension of proteins and are therefore relatively easily detectable, due to the size increase of the expressed proteins [29]. A recent systematic analysis identified a total of 59 human genes where non-AUG codons lead to 5′ extensions of known ORFs [30]. Both ACG and CUG have been described as alternative start sites [31], [32], [33]. However, the detection of non-canonical start sites, which are not in frame with the main ORF, is rather unlikely, as the translational product of such an uORF will often go unnoticed by conventional methods. Thus, given the high incidence of long and highly structured 5′UTRs in mRNAs encoding regulatory proteins in eukaryotes, it is likely that more non-conventional start sites remain to be discovered.

What could be the role of this mechanism with respect to synthesis of the Shank1 protein? Postsynaptic Shank1 levels are regulated by the translational regulator FMRP [22], and inappropriate levels of Shank are believed to be associated with mental disease in humans [19], [21]. Recent work showed that Shank1 is incorporated into PSDs later than the other Shank isoforms [34]. This may be due to delayed synthesis of the protein in dendrites. The Shank1 mRNA is abundant in dendrites [15], [16] whereas most neuronal mRNAs are restricted to cell bodies. Though we did not observe an effect of the 3′UTR on translational efficiency here, it should be noted that the 3′UTR carries the dendritic targeting element. Localization in dendrites might affect the translation rate of the Shank1 mRNA. Selection of start codons in uORF containing mRNAs is affected by the availability of eIF2-GTP-tRNA_i_^met^ ternary complexes, which is reduced by phosphorylation of eIF2α [35]. However, we did not observe alterations in translation of Shank1 reporter mRNAs in response to cellular stress, which induces phosphorylation of eIF2 (Katrin Studtmann, unpublished observations). Currently it is unclear whether the availability of ternary complexes in dendrites differs from that in neuronal cell bodies.

We currently assume that the stable secondary structure of the 5′UTR inhibits synthesis of Shank1 during transport of the mRNA from the cell body to its dendritic destination. Somatic Shank1 synthesis might cause problems as Shank1 tends to aggregate in the absence of postsynaptic partners [36]. In addition, Shank1 may induce postsynaptic complex formation [17], which should not occur in neuronal cell bodies. Further work will need to address how the mechanisms described in Figure 7 allow for translation of the mRNA in dendrites.

## Materials and Methods

### Ethics Statement

Work with animals was restricted to the preparation of tissues after sacrificing animals according to local regulations of the state authorities in Hamburg, Germany. Anaesthetized animal were killed by decapitation. Mice were bred in the Experimental animal facility, University Hospital Hamburg-Eppendorf, for purposes of this study. These experiments were registered with “Amt für Gesundheits- und Verbraucherschutz” of “Behörde für Soziales, Familie, Gesundheit und Verbraucherschutz”, State Government of Freie und Hansestadt Hamburg (Registration number Org 371).

### Mouse cortical neurons

Shank1 deficient mice have been described previously [37]. For genotyping, genomic DNA from tail biopsies was analyzed by PCR using forward primer CTGTAGTGTGTAGTGTTCCGACCTCC and reverse primers CCATCCACCCATCCATTCAGC (wt allele; 239 bp) and GCTACTTCCATTTGTCACGTC (ko allele; 430 bp). Cortical neurons were prepared from newborn (P1) animals and cultured for two weeks as described [38]. Cell lysates were prepared in RIPA buffer and analyzed by Western blotting.

### Antibodies

A rabbit antiserum directed against the Shank1 PDZ domain (recognizing all Shank variants) has been described previously [15]. For generation of antisera against protein fragments derived from the 5′ region of the Shank1 mRNA, corresponding cDNA sequences were cloned into pGEX vectors in frame with the glutathione-S-transferase (GST) coding sequence. GST fusion protein was expressed using standard methods, and used for custom immunization of rabbits (Biogenes GmbH, Berlin, Germany). Anti-monomeric red fluorescent protein (mRFP) has been described before [24]. Anti-neomycin phosphotransferase was obtained from Upstate/Biomol (Hamburg, Germany); anti GluR1, anti-eIF2a and phospho-eIF2a from Abcam Cambridge, UK; anti-β3 tubulin, anti-Shank1 and anti-MAP2 were from Sigma.

#### Expression constructs

A human cDNA fragment containing the 3800 nt of the 5′ region of the Shank1 cDNA was obtained by screening a phage cDNA library from human thalamus ([15]; see accession numbers AF163303 and EU872208). The fragment was cloned into the pcDNA3 expression vector via EcoRI sites. The EGFP coding sequence in pEGFP-N1 was replaced by the mRFP cDNA ([39]; kindly provided by Roger Tsien, Univ. of California, San Diego, CA) to generate pmRFP-N1 [24]. Parts of the Shank1 5′UTR were subcloned into pmRFP-N1, 5′ to the mRFP coding region. Site-directed mutagenesis was performed by PCR; as the high GC content of the 5′UTR made amplification impossible in case of the mutagenic primers for uAUG3, two fragments were amplified. One fragment located 3′ to uAUG3 was produced using the desired mutagenic primer, which contained an additional BamHI site, in combination with a primer derived from the vector. A second fragment containing the region 5′ to uAUG3 was amplified with a reverse primer containing a BglII site, and an appropriate vector derived primer. After restriction, fragments were recombined at the compatible BglII/BamHI sites thereby generating full length 5′UTR with the AUG3 mutation and an additional mutation which was due to the generation of an artificial BglII site. The identity of all constructs was verified by sequencing.

### Luciferase assays

cDNAs encoding WT and mutant human Shank1 5′UTR sequences were inserted upstream of the Photinus luciferase coding region into pBL [24]. After linearization using BamHI, capped RNAs were transcribed *in vitro* using T7 RNA polymerase (mMessage Machine kit, Ambion, Huntington, UK). Hundred ng of RNA were translated *in vitro* using rabbit reticulocyte lysate (Promega; Mannheim, Germany); luciferase activity was determined using the luciferase assay system (Promega), and a Berthold luminometer (Bad Wildbach, Germany). Bicistronic vectors derived from pBicFire [40] were transfected into HEK 293 cells or primary cultured cortical neurons using the calcium phosphate method. Cell lysates were sequentially assayed for *Photinus* and *Renilla* luciferase activities using the dual luciferase assay system from Promega.

### Peptide pulldown

A synthetic peptide corresponding to the C-terminus of GKAP/SAPAP1 (sequence IYIPEAQTRL) was obtained from Genemed Synthesis (San Antonio, USA) and coupled to NHS-activated sepharose (GE Healthcare) at a concentration of 3 mg/ml matrix. PDZ domain containing proteins were expressed in human embryonic kidney (HEK293) cells by calcium phosphate transfection of expression vectors. Cells were lyzed in RIPA buffer, followed by centrifugation at 20.500× g. PDZ fusion protein was precipitated from cleared lysates using the immobilized peptide and analyzed by SDS-PAGE. Gels were stained with Coomassie Brilliant Blue; bands were cut out, digested with trypsin, and analyzed by mass spectrometry as described [25].

### Bioinformatic analysis

Analysis of potential translation initiation sites was performed using NetStart 1.0 (available at http://www.cbs.dtu.dk/services/NetStart/) and ATGpr (available at http://flj.hinv.jp/ATGpr/atgpr/index.html). To identify potential non-AUG start sites, all CUG and ACG codons in the Shank1 5′UTR were changed to AUG first, as these programs only recognize conventional start sites.
